# Present and Future of the Poor-Law Infirmary

**Published:** 1913-10-25

**Authors:** 


### PRESENT AND FUTURE OF THE POOR-LAW INFIRMARY.

### The Infirmary—The Resident Medical Officer's Point of View.

By H. B. M.

The record of an infirmary resident, though containing nothing atypical or extraordinary, may prove of some interest. The position is an interesting one. The intricacies of the Poor Law, the high pressure of his daily routine work, the usual psychological study of institutional life with its whispered gossips and subterranean slanders—all have a fascination for the keen student of medicine and Nature. Apart from his personality and temperament, the experiences depend to some extent on his choice of infirmary, or its choice of him. The Guardians have a rather exalted idea of the position which they so generously remunerate at a princely £120 a year, plus the usual emoluments. The young medical stranger, after his expensive undergraduate career and, perhaps, gratuitous service in a provincial hospital, has usually to start at the lowest rung of the Poor-Law ladder.

The writer was, for nearly two years, the junior resident medical officer at one of the most recent and modern of Metropolitan infirmaries. The institution was, on the whole, fairly well equipped; but the microscope was the medical superintendent's; there was no sphygmomanometer or hæmocytometer, and until latterly no facilities for microscopical work of any kind. Owing to the medical superintendent's love of surgery the institution was, in this respect, quite up to modern standards.

Even had the institution been fully equipped, there would have been little or no time for technical bacteriological work or clinical finesse. Our records of cases were of the briefest; and when, on occasions, one attempted a reasonable record, one had to expect some chaff about the effort.

There were three medical men for an institution of 612 beds. The medical superintendent—whose time was occupied with administration, and surgical and revising work, and the two others, the senior and junior resident medical officer, between whom was divided the rest of the work, more or less equally, were kept busy with clinical work; these two latter were nominally allowed every alternate afternoon and evening for leisure, the resident on duty, as is customary, doing the whole work.

There were two days a week for surgical work in the theatre; these were the days on which the junior resident did duty. Theatre work occupied from 9.30 or 10 A.M. to 2, 3, 4, or even 5 P.M., depending on the gravity of the cases and the operations; the ward rounds, which usually occupied from one to two to three hours, had then to be done; and cases had to be received numbering 5 to 30 a day. Frequently on theatre days the junior had to rush off between or during operations to attend urgent calls in other parts of the building (say, to a sudden and grave hæmoptysis in a phthisical case) or to receive accident or police-ambulance cases in the receiving room. He was kept continually on the rush from morning till night. Meals frequently interrupted; telephone calls from sisters; minor administrative points to settle; night calls, varied, sometimes very frequent, three to four times a night; the night superintendent, however, was a woman of excellent judgment, and one could rely on her calls being absolutely necessary; cases to receive during the night were of common occurrence, some cases needing much treatment, or even immediate operation; urgent cases at all hours of the day, eclampsias for immediate delivery, police cases—all were of frequent, almost daily, occurrence. In fact, the resident had to work as hard as the resident in a voluntary hospital, with less thanks; the stay in a hospital is for the purpose of gaining experience and tuition at the hands of specially trained men. The sweating of the general practitioner compares favourably with the sweating of the indoor Poor-Law resident.

An *x*-ray plant was installed during my residence. The radiography was delegated to the dispenser, possibly owing to the medical officers being, perhaps, too busy otherwise. The dispenser had had no previous experience of *x*-ray work, but after some months some creditable work was done.

The medical superintendent though young was a man of parts. Had circumstances been favourable he would have risen to a great height in his profession as an exceptionally capable surgeon. His attitude towards the patients was admirable, gentle or firm as occasion demanded. Though often harassed and overworked, he was always courteous, and especially so to his medical subordinates. He was, perhaps, too deferential to the matron, and a firmer attitude in this direction would at times have redounded greater to his credit. He was very generous as to "off-duty" time; always willing to remain on duty himself, very frequently often insisting that one should go out for one's health. This is a very rare attitude for a medical superintendent to adopt. His generosity was also seen in the matter of extra fees. Unlike most infirmaries, the inquest fees were here shared with the resident who received the case, even though, as sometimes happened, the *post-mortem* examination had to be done by himself.

The nurses were of a mixed class, but the majority were quite well educated, showing the good judgment and choice of the matron. The sisters were excellent, the two theatre sisters of my tenure of office exceptionally so, and up to the standard of the voluntary hospitals. In fact, one of the theatre sisters, infirmary trained, is now theatre sister of one of the best-known gynæcological voluntary hospitals in London.

The matron was new to Poor-Law work. Every Poor-Law matron, owing to the gross understaffing of the institution medically, has to train herself to be ready for any emergency. Many act as assistant to the surgeon at all operations; some may even have to give the anæsthetic. This fact marred her administration. It was the virtue of keenness converted into a vice of interference. It was a desire to be able to fill the shoes of any absentee comfortably. But such a pose led to interference all round, to the great annoyance and discomfort of her staff. Ward arrangements were upset, and orders countermanded; she tried to be present at the carrying out of every treatment and of every birth, even when it was obvious that her assistance was not needed, and her presence a disturbing factor.

On the whole, the medical officer's position was a pleasant one; the etiquette of the institution was nearly perfect. The work was very hard, and the pay small; yet there were twenty-one months of much happiness, and of great medical, surgical, and sociological interest. In this infirmary chronic cases only accounted for about 25 per cent. of the inmates. Infirmary officers are often blamed for wrong diagnoses, thought to be due to carelessness or indifference. Before such a decision it is well to remember that there are infirmaries—and infirmaries; and that the resident medical officer, though willing, has not time to devote himself to great finesse of diagnosis in difficult and doubtful cases. His great object is the alleviation of pain and suffering.

